# Re-implantation after insufficient primary 125-i permanent prostate brachytherapy

**DOI:** 10.1186/1748-717X-8-194

**Published:** 2013-08-06

**Authors:** Paul Martin Putora, Ludwig Plasswilm, Wolf Seelentag, Johann Schiefer, Patrick Markart, Hans-Peter Schmid, Daniel Engeler

**Affiliations:** 1 Department of Radiation Oncology, Kantonsspital St. Gallen, 9007 St. Gallen, Switzerland; 2 Department of Urology, Kantonsspital St. Gallen, 9007 St. Gallen, Switzerland

**Keywords:** Prostate cancer, Brachytherapy, Re-implantation, Salvage, LDR, Seeds

## Abstract

**Introduction:**

We describe five patients receiving a re-implantation (RI) after post-operative dosimetry of the primary 125-I permanent prostate brachytherapy (BT) for prostate cancer revealed an insufficient dose coverage.

**Materials and methods:**

Five out of 222 consecutive patients treated (from March, 2001 to August, 2012) with 125-I BT, received a RI after dosimetric verification by CT and MRI fusion four to eight weeks after implantation displayed an insufficient dose coverage. RIs were performed with 10 to 19 seeds, three to four months after primary intervention. Dosimetry after RI showed an improved and sufficient total dose coverage in all patients.

**Results:**

At last follow-up (18 to 99 months, median 57 months), none of the patients had relevant implant associated side-effects. Functional outcome was comparable to patients after one-time implantation. PSA levels post intervention showed a decreasing tendency in 4 patients. One patient had a local recurrence after 12 months.

**Conclusion:**

In our series, approximately 2% of the patients treated with permanent prostate BT required a RI due to insufficient dose coverage. None of the patients who underwent RI experienced complications. Our series, although only with 5 cases and limited follow-up, along with other published reports, demonstrates good tolerability.

## Introduction

Prostate cancer (PC) is the most common solid neoplasm and the second most common cause of cancer death in men. Due to the increasing use of prostate-specific antigen (PSA) testing, the diagnosis of PC is rising worldwide. Most the new cases are diagnosed early with low PSA and Gleason scores. For these patients, permanent seed prostate brachytherapy (BT) is a treatment option associated with low morbidity and similar oncological outcome as with radical prostatectomy or external beam radiation therapy [1-3]. For the oncological long-term success of BT, it is important to ensure the prostate is covered with sufficient dose. Prostate cancer foci occurring in areas with insufficient dose may lead to recurrence. The most frequently used parameters to assess quality of implantation in BT are the volume of the prostate covered by 100% of the prescribed dose (V100) and the dose covering 90% of the prostate volume (D90) [4]. The decline of dose coverage between intraoperative planning and post implant dosimetry is a known phenomenon. For permanent 125-I seed implantation, loose or stranded seed methods can be used. As published by a Dutch group, the stranded seed method seems to have a higher rate of dose decline. The compared methods were RAPID strand, Intersource strand and loose selectSeeds, the decline of D90 was 40 Gy, 25 Gy and 15 Gy on average [5].

There are several reports linking postimplant dosimetry to biochemical outcome. The first report showing higher biochemical relapse rates for patients with D90 of <140 Gy was by Stock & Stone [6]. The American Brachytherapy Society suggested that implants should meet the D90 >130 Gy dosimetric criterium [7] because lower doses are associated with an increased risk of oncological failure [8]. Whereas the criteria for excellent postimplant dosimetry are well accepted, the dose limits that should prompt salvage treatment by external beam radiotherapy or reimplantation (RI) are not clear. In general, permanent prostate seed BT is a well tolerated treatment, but evaluation of critical organ dosimetry and associated functional results is mandatory. If a RI is planned, toxicity caused by the second intervention may be an issue and must be considered. Because of sparsity of reports on RI, we herein describe our experience with five patients receiving a RI after dosimetry of the primary 125-I permanent prostate BT revealed insufficient dose coverage.

## Material and methods

### Patient selection for permanent seed prostate brachytherapy

At our institution, patients are eligible for permanent seed 125-I prostate BT as a monotherapy if they are diagnosed with localized low-risk prostate cancer (PSA < = 10 ng/dl, Gleason score < =6, clinical stage T1 to T2a), or occasionally with intermediate prostate cancer (either PSA 10-20 ng/dl, Gleason score 7, or clinical stage T2b).

For the application several seed types were used: Rapidstrand (first patient described here), IsoCord S06 (second patient) and IsoCord S17 (last three patients), respectively. Seed activity used is typically in the range 0.70 to 0.78 mCi/Seed, depending on the size of the prostate. The activity is chosen to result in approximately 40-60 seeds for the implantation.

Patients up to 75 years of age are treated depending on their individual life expectancy. Patients with prostate volumes >60 ml undergo preoperative downsizing with 3 months of hormonal therapy using Luteinizing-hormone-releasing hormone agonists.

### Patient evaluation and follow-up

Besides oncological parameters including digital rectal examination, PSA, transrectal ultrasound, biopsy Gleason grade, staging examinations (computed tomography of the pelvis and bone scan) clinical functional data were recorded routinely. They included residual urine volume, maximum urine flow rate (Q-max), international prostate symptom score (IPSS), and IPSS-quality of life (IPSS-QoL) [9]. Genitourinary, gastrointestinal and musculoskeletal adverse events were recorded according to the National Cancer Institute common toxicity criteria (CTCAE v3.0) [10]. Follow-up examinations were scheduled at six weeks, six months, one year, and yearly thereafter. The median follow-up was 57 months (ranging from 18-99 months). For comparison of micturition and quality of life data from patients treated at our institution with primary implantation only were compiled (see Table 1).

**Table 1:** Micturition parameters baseline and after treatment (mean values and range)

**Number of patients RI / control**	**Baseline 5 / 137**	**6 weeks 5 / 136**	**6 months 5 / 142**	**1 year 4 / 130**	**2 years 3 / 115**	**3 years 2 / 90**
Residual urine (ml) Control:	0 [0-0] 23.3 [0-300]	48 [20-80] 55.8 [0-900]	58 [20-90] 39.8 [0-400]	32.5 [0-100] 24 [0-200]	18 [6-30] 16.1 [0-122]	0 [0-0] 17.4 [0-200]
Residual urine (ml/s) Control:	20.8 [16.1-32] 18.5 [5.5-40]	15.2 [10.3-18.8] 11.1 [7-26]	11.6 [9.4-14.1] 12.6 [3.3-35]	16.3 [13.5-19] 14.6 [4.1-35.7]	20.4 [18.4-24.1] 16.1 [3.8-40]	19.8 [17.4-22.1] 15.1 [2.2-34.8]
IPSS Control:	5.7 [3-11] 5.8 [0-22]	11.7 [7-19] 15.3 [1-34]	14.3 [11-19] 12.7 [0-34]	12.3 [7-15] 10.3 [1-26]	9.0 [6-12] 9.5 [0-26]	6.5 [4-9] 7.5 [1-28]
IPSS-QoL Control:	0.7 [0-1] 0.96 [0-5]	2.7 [1-4] 2.6 [0-6]	2.5 [1-4] 2.2 [0-6]	2.3 [1-4] 1.7 [0-6]	2.0 [1-3] 1.5 [0-5]	2.0 [1-3] 1.2 [0-5]

As control, mean values of 144 consecutive patients treated without re-implantation are shown. The first line shows the values for the re-implanted patients, the second the control group consisting of patients not receiving a re-implantation. (IPSS data was available for 3,3,4,3,2,2 patients respectively).

### Implant planning and procedure

The method implemented was an intraoperatively planned template-guided transrectal ultrasound-guided permanent 125-I BT. This method is established and has demonstrated excellent prostate dosimetry results and rectal sparing [11].

### Implantation procedure

The prostate structure was defined in ultrasound images by the urologist. The planning procedure is then a multidisciplinary process involving the urologist, radiation-oncologist and medical physicist. The preplan is used only to determine the approximate number of seeds required. At the day of the implantation the ultrasound probe and template are fixed and ultrasound images are aquired. Based on these the prostate is contoured and a plan calculated. The prescribed dose is 145 Gy to the prostate. The dosimetric criteria were V100%prostate ≥ 95% of the prostate structure volume. The maximal urethral dose was aimed to be kept less than 150% of the prescribed dose. The volume of the rectum receiving 100% of the prescribed dose was aimed to be kept under 0.3 cm^3^. The seeds were implanted through the template under biplanar ultrasound guidance, additionally longitudinal placement is verified by X-ray; only in rare cases of deviations during implantation the plan needs to be adapted in real-time. On the same or next day of implantation a conventional x-ray anterio-posterior is performed for immediate documentation.

### Postimplantation dosimetry

Six weeks (on average) post implant MRI and CT scans of the pelvis were acquired. The dose distribution is calculated on MR-CT fusion images to determine whether the coverage of the prostate is satisfactory, as recommended by the American Brachytherapy Society [7].

### Decision criteria for reimplantation

Decision criteria were based on a case-to-case basis determined by insufficient dose coverage (D_80_ below 100% or V_100_ below 80%) and patient preference. As the reimplantation is not considered a standard procedure, only larger deviations from the expected dose coverage would warrant a reimplantation, this is why the D80 was used as parameter instead of D90.

### Salvage procedure

The salvage re-implantation procedure is prepared based on the postimplant MR and CT scans. The distribution of the additionally needed seeds (same activity as in the primary seed application) is then transferred to the template positions intraoperatively to cover the cold spots. Six weeks after re-implantation CT and MRI scans are performed again for dose calculations. The time gap is not incorporated in dose calculations. The dose coverage goals and consideration of the organs-at-risk were basically the same as in the primary implantation.

## Results

Out of 222 consecutive patients treated (from 2001 to August 2012) with 125-I permanent prostate BT in five patients (2.3%) RI was performed. In all five patients the RI was performed three to four months after the primary intervention. We performed a detailed review of all five cases including their dosimetric parameters, oncological and functional follow-up.

### Summary of all reimplanted patients

All patients undergoing salvage RI had localized low-risk prostate cancer at diagnosis (Table 2). No relevant differences in terms of residual urine, Qmax or IPSS and IPSS-QoL could be observed in the RI group when compared to the control group (217 patients without RI) (Table 1).

**Table 2:** Basic oncological parameters of patients undergoing reimplantation

**Pt. No.**	**Age (years)**	**T stage**	**Gleason score**	**Init. PSA (ng/ml)**	**Nadir PSA (ng/ml)**	**F/U (months)**	**Last PSA (ng/ml)**
1	45	T2a	4(2 + 2)	6.5	0.18	99	0.18
2	54	T1c	6(3 + 3)	6.7	0.03	80	0.03
3	53	T1c	6(3 + 3)	3.68	0.02	57	0.02
4	67	T1c	6(3 + 3)	12.8	2.07	43	10.1
5	60	T1c	6(3 + 3)	5.09	1.33	18	1.33

The minimal dose to 5% of the urethra volume (D5u), representing the maximum dose to urethra, was similar to patients without a re-implantation. To put this into perspective, intra-operative dosimetric data of the last 166 patients were retrieved from our database. The average D5u of patients receiving a brachytherapy for prostate cancer was 143% (ranging from 126% to 177%), the median being 137%.

The rectal volume receiving at least 100% of the prescribed dose for patients undergoing a re-implantation is also described in Table 3. The same 166 patients were evaluated. The average for patients not undergoing a re-implantation was 0.22 (range from 0 to 2.7), median 0.26 cm^3^.

**Table 3:** Maximal urinary and rectal toxicities as well as corresponding dosimetric parameters

	**D5u (%)**	**Maximal urinary toxicity**	**Timepoint of toxicity**	**V100r (cm**^**3**^**)**	**Maximal rectal toxicity**
Patient 1	150.0	0	-	0.1	0
Patient 2	161.4	frequency, grade 1	2 years	0.3	0
Patient 3	131.1	pain, grade 1	6 months	0.3	0
Patient 4	129.9	0	-	0.3	0
Patient 5	153.7	retention, grade 1	6 months	0.9	0

V100r – the rectal volume receiving at least 100% of the prescribed dose. D5u – the minimum dose to 5% of the urethra volume.

The objective parameters and IPSS quality of life scores showed a temporary worsening and a trend towards normalization after approximately one to two years. This is a typical observation in brachytherapy for prostate cancer and not specific for RIs.

At 6 weeks after reimplantation none of the patients experienced any toxicity. At 6 months patient 3 experienced genitourinary pain grade 1 and patient 5 urinary retention grade 1. At one year both normalised (grade 0). Patient 2 experienced urinary frequency/urgency grade 1, which also normalised by the 2 year follow-up. Other toxicity was not observed.

The number of seeds used for primary as well as secondary implantation together with the dosimetric parameters of the five patients after primary implant and RI are described in Tables 4 and 5. Single seed migration is not enough to cause a significant underdosage requiring a RI procedure. In all 5 cases where a RI was conducted the underdosage was caused by multiple seeds. Of the last 166 patients consecutively treated and digitally documented a total of 6 patients had a primary implantation that would qualify for a re-implantation. Of these in two cases a re-implantation was not considered appropriate, the other four received a re-implantation. Based on this we would estimate the rate of ‘unacceptable’ implantations to be about 1 in 25.

**Table 4:** Number of seeds used and volume of the prostate measured postoperatively

**Patient number**	**Seeds used at implantation, in brackets the seeds present at evaluation (N)**		**Prostate volume measured by ultrasound (ml)**	
	**Primary**	**Secondary**	**Primary implantation**	**Reimplantation**
1	50	12	34.9	37.2
2	56	16	31.5	30.8
3	51 (48)	19 (17)	40.3	40.8
4	42	12	43.8	31.7
5	49	10	40.0	42.5

**Table 5:** Dosimetric parameters after primary (p) and secondary (s) implantation

**Patient number**	**D90p [%]**		**V50 [cm**^**3**^**]**		**V100 [cm**^**3**^**]**		**V150 [cm**^**3**^**]**		**V100p [cm**^**3**^**]**		**V150p [cm**^**3**^**]**	
	p	s	p	s	p	s	p	s	p	s	p	s
1	62.8	104.4	106.5	126.4	52.6	62.7	32.6	37.6	25.5	34.1	19.5	25.5
2	67.6	140.2	160.9	215.6	76.7	106.9	45.8	66.4	23.4	30.7	16.1	26.3
3	57.2	123.5	112.7	161.2	55.4	82.4	32.9	51.0	27.5	39.4	19.0	32.2
4	77.0	107.9	110.5	132.5	47.7	65.0	20.1	40.1	32.4	29.3	14.6	22.9
5	61.7	95.9	130.1	162.5	59.8	81.7	31.7	42.7	26.4	37.4	13.3	19.5
Median control	105.4		142.7		70.5		41.7		36.9		27.3	

(D90p – the percentage of the prescribed dose covering 90% of the prostate volume, V50, V100, V150 – the volume receiving 50%, 100% and 150% of the prescribed dose respectively, V100p, V150 – the volume of the prostate in cm^3^ receiving 100% and 150% of the prescribed dose respectively).

The median values of a control group of 165 consecutive patients was added as control.

**Figure 1:**
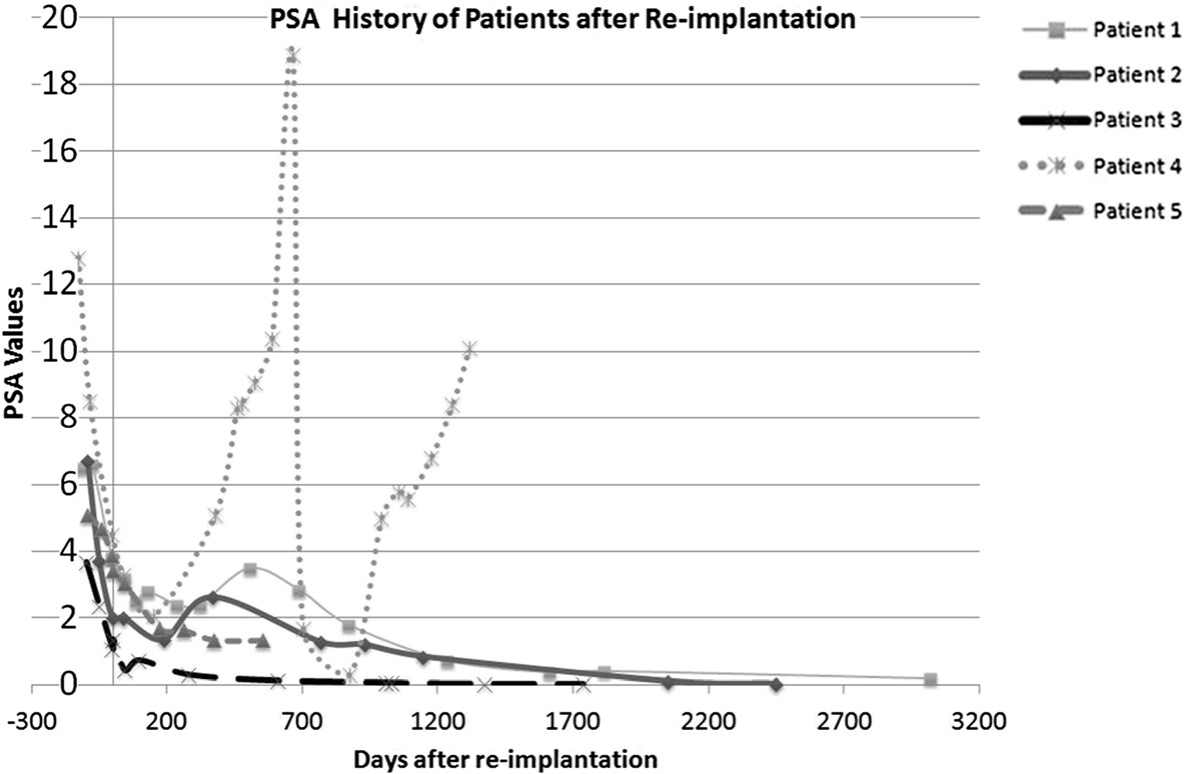
**PSA history of 5 patients undergoing prostate seed re-implantation (RI).** Patient number 4 had a recurrence at 22 months, whereas all other patients had no biochemical relapse.

The PSA values of the patients are shown in Figure 1. Two patients experienced a bounce phenomenon, a transient increase in PSA value after brachytherapy. Patient number 4 experienced a biochemical failure.

### A sample case

To demonstrate the problem a sample case is described here (patient No. 3 in tables). The underdosed prostate values were caused by a shift of the implanted 48 seeds caudally from the planned position, additionally 3 seeds were lost (Figure 1). The re-implantation was performed with 19 seeds, however two were lost here as well resulting in 17 re-implantation seeds. The dose coverage in the postoperative CT/MRI fusion images of the primary implantation was clearly insufficient in this situation (D90 57.2%, D80 75.98%), thus the decision for RI was taken. The resulting dose distribution was significantly better after RI (D90 123.5%). The reason for the massive shift remains unclear as the ultrasound and guided placement was as inconspicuous. To put this into perspective, of the last 161 consecutive patients (including the last three re-implantations) seed loss was detected in 34% of patients. Of these 13% had single seed loss, 19% had 2-4 seeds lost and in three cases 5, 9 and 10 seeds were lost respectively.

### Recurrence

**Figure 2:**
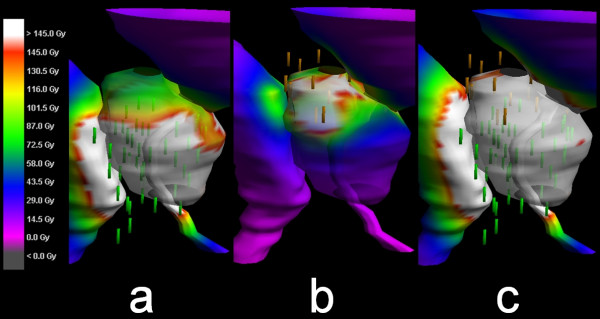
**The surface dose to the prostate (color wash) shows the areas of doses under 145 Gy with non-white colors.** The surface doses of insufficient primary (48 seeds after loss), and isolated secondary seed placement (17 after loss) are shown in panels **(a)** and **(b)**, respectively. The resulting total dose indicates a good dose coverage of at least 145 Gy (white) throughout the prostate surface **(c)**.

In one out of the five patients (Patient number 4), after reaching a nadir of 0.5, a recurrence was detected 22 months after salvage RI. The recurrence was detected by a PSA rise (up to 18.9 ng/ml). The recurrence rate at our institution for patients treated with primary iodine seed brachytherapy was 6.8% at 5 years based on patient data with at least 2 years of follow up (195 evaluable patients). A 18 F-choline PET/CT was performed, where a solitary choline uptake could be identified amidst artifacts from brachytherapy seeds. The dosimetric parameters of the patient were satisfactory after RI. The specific location of recurrence could not be identified as underdosed on retrospective review. Based on patient preference and pre-irradiation further surgery or radiotherapy was refrained from. After intersdisciplinary discussion the decision was taken to perform a High Intensity Focal Ultrasound (HIFU) ablation of the prostate, additionally to this an androgen deprivation therapy was performed with an LHRH agonist for 6 months. As a result of this the PSA decreased to 0.3 ng/ml but has gradually been rising again to over 10 ng/ml after stopping hormonal therapy. A bladder neck resection was performed and a transurethral resection of the prostate including bladder stone lithotripsy. The resulting histology of the prostate was without proof of malignant cells. Currently (5 years after re-implantation) the patient is asymptomatic and being observed without further therapy (See Patient No. 4, Figure 2).

## Discussion

Post-implant dosimetry is essential to determine the outcome of prostate seed brachytherapy [6]. Urethral toxicities are feared with doses exceeding 300 Gy. The concern of toxicity hinders many in deciding to perform an immediate salvage therapy. In our series of 222 patients we performed a RI in 5 patients where the dose coverage was considered unacceptable and a RI clinically feasible. Many brachytherapy series do not report grave underdosing; therefore it is difficult to establish how often this phenomenon occurs in practice.

Algorithms to plan RI have been designed and tested in phantoms, their routine use in practice is not yet implemented widely to our knowledge [12]. A single case demonstrating the feasibility was published in 2005 [13]. The cause was a systematic source placement error that left the base of the prostate significantly underdosed. In this report a RI increased the percentage of volume receiving 100% of the prescribed dose (V100) from 46% to 98% and the dose to 90% of the Volume (D90) from 49 to 201 Gy. In the described report, the urinary morbidity was increased and was relieved by medication. Alternative methods involving robotic seed placement have also been described (in primary implantation) [14].

Another report of seven cases of RI with 125-I prostate brachytherapy after insufficient initial postimplant dosimetry reported an optimal dosimetric outcome. The short-term PSA follow-up was favorable. The authors conclude however that the ultimate benefits and long-term toxicity remain unknown [15].

Due to a lack of know-how and published evidence a decision is often not easy to take. In the 5 cases at our institution, based on the post-implant dosimetry, we decided to perform a salvage RI. The procedures were not associated with any relevant side-effects. This of course needs to be interpreted with caution. The therapy was tolerated well. Patients did not experience higher urinary toxicity than expected after a regular implant (Table 3). Alltogether, the re-implantations were well-tolerated.

In our series rectal toxicity was not observed (CTCAE Score 0); interestingly in the series reported by Keyes et al. the median VR100 and VR50 (representing the rectal volume receiving 50% or 100% of the prescribed dose respectively for the re-implant patients was lower compared to the patients with successful first implants at their institution [15].

As insufficient dose coverage is a rather rare event and not all of these will undergo RI, the resulting absolute numbers are low. Linked with the rare occurrence, there is sparse literature on the subject. Based on this fact it is currently impossible to establish evidence based recommendations for RI after insufficient primary 125-I prostate brachytherapy.

## Conclusion

In our patient collective 2,3% (5 of 222) of patients treated with permanent prostate BT required a RI due to insufficient dose coverage. Patients undergoing RI after 125-I seed BT did not experience relevant treatment associated side effects up to this day. There are only few published reports on RI and longterm data are still missing. Conclusions on local control are not statistically significant, due to the low number of 5 patients, including one local failure. Our series, along with other published reports, however, demonstrated good tolerability. For individual patients a RI should be discussed after insufficient primary 125-I seed brachytherapy for low-risk prostate cancer.

## Competing interests

The authors declare that they have no competing interests.

## Authors’ contributions

DE, HPS, PM and LP were primarily involved in treatment. DE managed the database. JS and WS were responsible for the physics contribution. Data retrieval, analysis was performed by PMP, DE and WS. All authors contributed to the manuscript creation. All authors read and approved the final manuscript.
